# The Relationship between the Disability Prevalence of Cataracts and Ambient Erythemal Ultraviolet Radiation in China

**DOI:** 10.1371/journal.pone.0051137

**Published:** 2012-11-30

**Authors:** Yang Wang, Jiaming Yu, Qian Gao, Liwen Hu, Na Gao, Huizhi Gong, Yang Liu

**Affiliations:** 1 School of Public Health, China Medical University, Shenyang, People's Republic of China; 2 Ophthalmology Department, The Fourth Affiliated Hospital of China Medical University, Shenyang, People's Republic of China; Duke University, United States of America

## Abstract

In Western countries, ultraviolet (UV)-induced skin cancer has been studied extensively regarding the high incidence of skin cancers in the white population; however, for people of color, cataracts are the main public health issue in relation to increased ambient ultraviolet radiation (UVR). To our knowledge, few studies have been conducted examining the relationship between cataracts and ambient UVR in China. In this study, we aimed to explore the relationship between and the factors influencing the disability prevalence of cataracts and annual ambient erythemal UVR exposure in 31 regions of China. The data used to determine the disability prevalence of cataracts was obtained from the Second China National Sample Survey on Disability. The regional annual erythemal UVR was calculated using Geographic Information System (GIS) methods based on data from the National Aeronautics and Space Administration (NASA) database. The relationship between the disability prevalence of cataracts and the annual ambient erythemal UVR was examined by using logistic regression. Both the age-standardized disability prevalence of cataracts (OR = 3.97, 95%CI 1.30–12.13, per 100KJ/m^2^ increase in annual ambient erythemal UVR) and the disability prevalence of cataracts among a population ≥65 years old (OR = 3.97, 95%CI 1.30–12.13, per 100KJ/m^2^ increase in annual ambient erythemal UVR) were higher in association with higher ambient erythemal UVR. Regions with higher urbanization and educational levels had lower disability prevalence of cataracts. We found positive associations of the age-standardized disability prevalence of cataracts and the disability prevalence of cataracts among a population ≥65 years old with ambient erythemal UVR in 31 regions of China.

## Introduction

Since the hole in the ozone layer in the South Pole was firstly reported in 1985 [1], ozone depletion has become an important environmental issue that has attracted worldwide attention. Two main public health issues in relation to ozone depletion, increased ultraviolet (UV)-induced skin cancer and cataracts, have been studied by researchers from different countries over the past two decades [2]–[8]. In Western countries, UV-induced skin cancer is more well studied than cataracts due to the high incidence of skin cancer among the white population. For people of color, however, cataracts have been the main public health concern due to increased ambient UVR [3]–[5], [9] because this population is less likely to develop skin cancer due to the protective properties of their skin.

The relationship between cataracts and ambient UVR or UV exposure has been established in previous studies [7], [10]–[12]. For example, West et al. reported that the odds of cortical opacity increased with increasing ocular exposure to ultraviolet radiation-B (UVB) [10]. Additionally, West et al. estimated that 167,000–830,000 additional cases of cortical cataracts would be identified by 2050 in the U.S. with 5–20% ozone depletion resulting in an increase of UVB rays [7]. However, to our knowledge, few studies have been conducted on the relationship between cataracts and ambient UVR or UV exposure in China.

The unique geographic condition and epidemiological characteristics of cataracts make China an ideal country to study the relationship between cataracts and ambient UVR. From a geographic point of view, from the southernmost region to northernmost region, China extends across nearly 50 degrees latitude, and the altitude of the western and eastern regions differs by nearly 4,000 meters. China's wide ranges in latitude and altitude provide a broad environmental gradient for estimating UV exposure.

In 2007, China's cataract patients numbered more than 60 million, and the population with a disability caused by cataracts numbered more than 7 million [13]. Today, cataracts are the leading cause of visual disability in China [14], [15]. When taken together, the enormous public health burden attributed to cataracts along with the country's geographic conditions make studying the relationship between the prevalence of cataracts and ambient UVR in China an urgent and suitable endeavor. Because up-to-date national data on the prevalence of cataracts was not available due to the lack of recent national epidemiological surveys, in the present study, we used the disability prevalence due to cataracts obtained from the Second China National Sample Survey on Disability as the index of UV-induced health effects on eyes.

The present study used data from the Second China National Sample Survey on Disability in 2006 to calculate the age-standardized disability prevalence of cataracts in 31 regions of China and used estimated daily ambient erythemal UVR from National Aeronautics and Space Administration (NASA) satellites, together with ArcGIS software, to calculate annual ambient erythemal UVR in corresponding regions. The main objectives of this study were (1) to estimate the disability prevalence of cataracts and the annual ambient erythemal UVR in 31 regions of China; (2) to evaluate the relationship between the disability prevalence of cataracts and annual ambient erythemal UVR in China; and (3) to compare the disability prevalence of cataracts and annual ambient erythemal UVR by latitude and altitude, respectively.

## Methods

### 1. Ethics Statement

The Second China National Sample Survey on Disability was a government-organized survey, and was conducted by the China Disabled Persons' Federation and approved by the State Council of the People's Republic of China. The procedures of this survey satisfied the requirements of the Statistics Law of the People's Republic of China. All survey subjects completed the written informed consent provided by the Chinese government.

### 2. Measurement of the disability prevalence of cataracts

**Figure 1:**
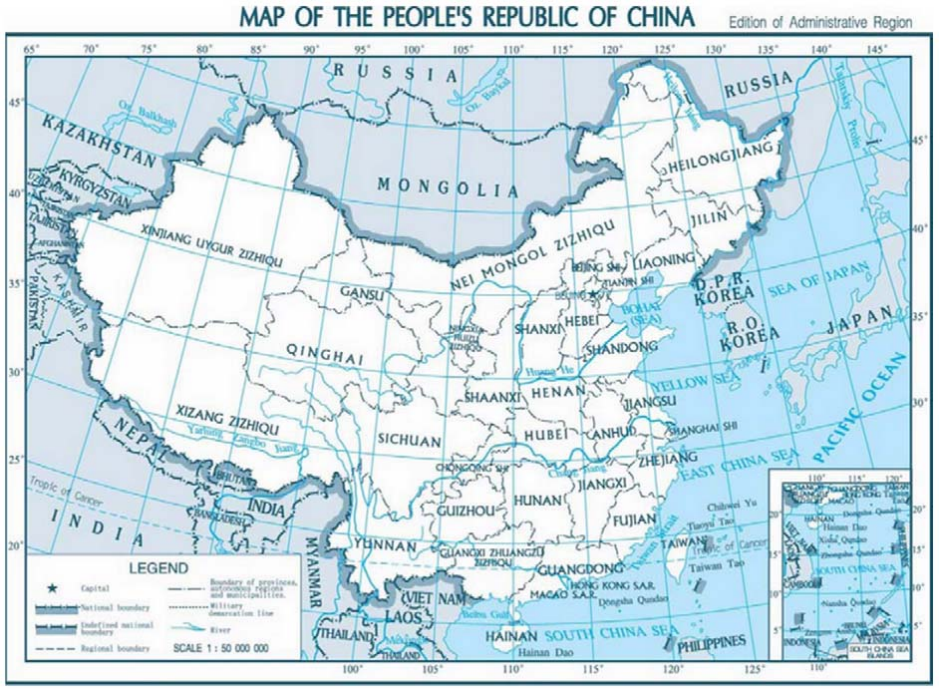
Map of China with the 31 regions included in the present study. Note: Taiwan, Hong Kong, and Macau were not included in the Second China National Sample Survey on Disability and thus were not included in our analyses. Cited from the National Administration of Surveying, Mapping and Geoinformation website, 2007. Standard map service. Available: http://www.sbsm.gov.cn/article/zxbs/dtfw// [Accessed 31 August 2011].

The Second China National Sample Survey on Disability was carried out on April 1, 2006 in 31 regions, including 22 provinces, 5 autonomous regions, and 4 municipalities in Mainland China. The geographic distribution of the 31 regions is shown in Figure 1. A stratified, multi-phased cluster random sampling design was applied in this survey. Sampling was conducted at three levels. The offices at the provincial level randomly selected 734 counties (cities or districts) based on economic development in each region. Each county then randomly selected 4 towns (with the exception of Beijing, Tianjin, and Shanghai, which selected more than 4 towns each) for a total of 2,980 towns (townships or streets). Finally, each town and street randomly selected 2 communities for a total of 5,964 communities represented in this survey [13]. The nationwide participation rate was 1.93‰. The investigation was conducted in two stages. The first stage consisted of residential family investigation. The 771,797 sampled families and 2,526,145 people within the included families were asked to report the disabled individuals who suffer from abnormalities such as a loss in function (psychologically or physiologically) or anatomical structure of a certain organ and whether they have totally or partially lost the ability to perform an activity in a way considered to be normal based on the guidelines provided in the screening table of the Second China National Sample Survey on Disability. The second stage involved the investigation of the identified disabled persons. All candidates received medical tests and were graded according to the Grading Standard of Disabilities of the Second China National Sample Survey on Disability. Of those selected, there were 142,112 families with disabled persons and 161,479 people with disabilities within the included families.

This survey targeted populations within the People's Republic of China who were permanent residents of the sampled residential quarters. Three types of survey forms were used: household questionnaires, disability questionnaires and community questionnaires. There were 52 survey items in all that included family and personal information on the sample group, focusing on the causes of disability, living conditions and major needs of the disabled population.

In this survey, “visual disability” referred to poor vision or the constriction of visual field in both eyes that may have been caused for various reasons and is uncorrectable. Visual disability consisted of two categories: blindness and low vision. The grading standard of visual disability is shown in Table 1 [13]. In the Second China National Sample Survey on Disability, “visual disability” was measured first and the reason of visual disability was examined by ophthalmologist for each visually disabled subject. If the subject was diagnosed as cataract, this subject was categorized as “visual disability due to cataract”.

**Table 1:** Grading standard of visual disability.

Category	Degree	Optimum corrected vision
Blindness	Degree 1	<0.02 (no light perception or visual field radius less than 5 degrees)
	Degree 2	0.02–0.05 (or visual field radius less than 10 degrees)
Low vision	Degree 3	0.05–0.1
	Degree 4	0.1–0.3

Cited from Documentation of the Second China National Sample Survey on Disability, 2007.

Notes: (1) Blindness or low vision refers to both eyes. In the event that the degree of the two eyes were different, only the eye with better vision was considered. If a person had one eye that was considered to be blind or to have low vision but the other eye was not recognized as visually disabled (greater than 0.3), then that person was excluded from this analysis. (2) The optimum corrected vision referred to the best vision after appropriate eyeglass rectification or vision measured by pinhole glass. (3) Persons with field of vision less than 10 degrees were recognized as visually disabled.

### 3. Measurement of ambient erythemal UVR

Erythemal UVR is commonly calculated by weighing the solar UV radiation with the erythemal action spectrum to reflect the erythemal effect of UVB radiation to the human skin. The estimated daily ambient erythemal UVR, expressed in joules per meter squared (J/m^2^), was obtained on a 1° of latitude (89.5S to 89.5N) by 1° of longitude (180W to 180E) grid for all of China. The grid was generated from the NASA Goddard Space Flight Center Data Archive Center database of readings from the Total Ozone Mapping Spectrometer mounted on the Nimbus-7 satellite [16] and Earth Probe satellite [17]. We estimated the erythemal UVR measurement by averaging daily estimates from 1980 through 2005 and calculated the annual erythemal UVR based on the assumption that the relative distribution of annual UVR was stable over time. As the NASA database did not provide a complete dataset of estimated daily erythemal UVR from 1993 to 1996, data from these years were not included in this study. ArcGIS 9.2 (http://www.esri.com/software/arcgis/index.html) software was used for spatial modeling of the erythemal UVR data, and the selection function was used to obtain region-level average daily erythemal UVR. Then annual erythemal UVR was calculated based on the regional average daily erythemal UVR.

### 4. Statistical analysis

We calculated the number of patients with visually disabling cataracts of different age groups in each of the 31 regions (0–14 years, 15–64 years and ≥65 years) based on the survey data. We calculated the total and age-specific disability prevalence of cataracts for each region by dividing the number of patients with visually disabling cataracts in each age group by the surveyed population in each age group.

To control for the effect of age on the disability prevalence of cataracts, we age-standardized the disability prevalence of cataracts using an direct standardization method. Several steps were included in this method: (1) the disability prevalences of cataracts for each of the three age groups (0–14,15–64, ≥65) were obtained from the surveyed population in each region; (2) the disability prevalence of cataracts in each of the three age groups in each region was multiplied by the number of people representing the standard population (the entire population of the survey) in each of the three age groups, and the three products were then summed to obtain the expected disability population of cataracts for each region; (3) the standard population was divided by the expected disability population of cataracts in each region to obtain the age-standardized disability prevalence for each region.

We also calculated the annual ambient erythemal UVR in three different decades (1980 s, 1990 s, and 2000 s) and the average annual ambient erythemal UVR of 31 regions in China.

SPSS 17.0 software was used to perform a logistic regression analysis to establish the relationship between the age-standardized disability prevalence of cataracts and the annual erythemal UVR. The age-standardized disability prevalence of cataracts was defined as “1” if it was larger than the median and as “0” if it was equal or smaller than the median, and it was analyzed as the binominal dependent variable. The annual erythemal UVR was the independent variable in the model. Similarly, the relationship between the annual ambient erythemal UVR and the disability prevalence of cataracts among a population ≥65 years old was established using the same method. In these two models, the unit of the annual ambient erythemal UVR was transferred from KJ/m^2^ to 100KJ/m^2^.

**Figure 2:**
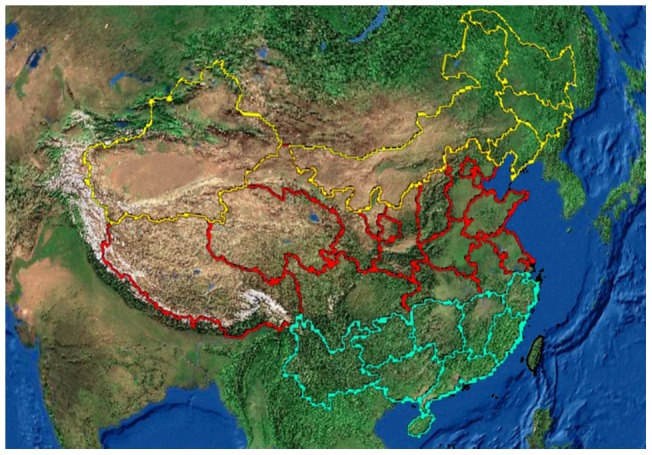
Illustration of the divisions of the three latitude groups. Note: Regions with green, red, and yellow borders are defined as latitude group 1, latitude group 2 and latitude group 3, respectively.

**Figure 3:**
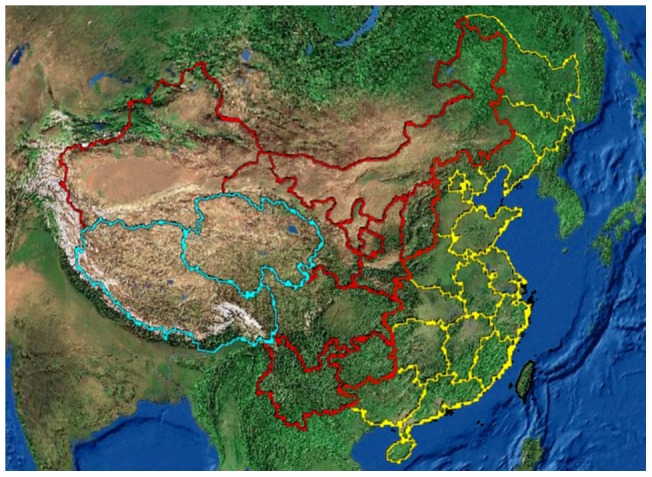
Illustration of the divisions of the three altitude groups. Note: Regions with green, red, and yellow borders are defined as altitude group 1, altitude group 2 and altitude group 3, respectively.

Thirty-one regions were divided into three groups based on latitude and altitude. Regions with an average latitude below or including 30°N were allocated to latitude group 1; regions with an average latitude between 30°N and 40°N were allocated to latitude group 2; and regions with an average latitude above or including 40°N were allocated to latitude group 3 (Figure 2). Regions are divided into three altitude groups based on the unique geographic feature of China, which Chinese geographers name “three-step ladder”. Regions with an average altitude higher or equal to 4,000 meters were allocated to altitude group 1; regions with an average altitude between 500 meters and 2,000 meters were allocated to altitude group 2; and regions with an average altitude less than 500 meters were allocated to altitude group 3 (Figure 3). Regions with an average altitude between 2,000 meters and 4,000 meters don't exist in China. Kruskal-Wallis test was used to compare the age-standardized disability prevalence of cataracts, disability prevalence of cataracts among a population ≥65 years old, and the ambient erythemal UVR among the three latitude groups and among the three altitude groups. Wilcoxon rank sum test was performed to do pairwise comparisons by adjusting α level to 0.017 (0.05/3).

To roughly reflect the effect of urbanization and educational levels on the amount of time people spent outdoors and further on the disability prevalence of cataracts, we compared the age-standardized disability prevalence of cataracts and the disability prevalence of cataracts among a population ≥65 years old between Beijing and Hebei as well as between Shanghai and Zhejiang. Beijing (longitude 115°24′E-117°30′E and latitude 39°28′N-41°05′N; altitude≈43 meters) and Shanghai (longitude 120°51′E-122°12′E and latitude 30°40′N-31°53′N; altitude≈4 meters) are two municipalities that are self-governed. These two cities have high population densities, high urbanization levels, and high education levels, resulting in a high proportion of indoor workers in these two cities. Hebei Province (longitude 113°04′E-119°53′E and latitude 36°01′N-42°37′N; altitude≈50 meters) and Zhejiang Province (longitude 118°24′E-122°24′E and latitude 27°30′N-31°00′N; altitude≈350 meters) are located at geographic positions where the latitude and altitude are similar to those of Beijing and Shanghai, respectively. The data in Table 2 showed that the annual ambient erythemal UVR in Beijing and Heijing were similar and that the exposure in Shanghai and Zhejiang were also similar. Compared to Beijing and Shanghai, the urbanization and education levels of the population in Hebei Province and Zhejiang Province were lower. In 2005, the proportion of the urban population in Beijing was 83.62%, whereas in Hebei province, the urban population was 37.69%. The proportion of the urban population in Shanghai was 89.09%, whereas 56.02% in Zhejiang Province represented the urban population [18]. The proportion of people with a college education or above was 12.13% in Beijing, but only 1.15% in Hebei. The proportion of people with a college education or above was 8.23% in Shanghai, but only 1.83% in Zhejiang [12]. We compared the age-standardized disability prevalence of cataracts and the disability prevalence of cataracts among a population ≥65 years old between Beijing Province and Hebei Province and between Shanghai Province and Zhejiang Province to observe the influence of outdoor time on the actual UV exposure among the population and its influence on the disability prevalence of cataracts.

**Table 2:** Disability prevalence of cataracts in 31 regions of China (sorted by the age-standardized disability prevalence of cataracts).

Region	Surveyed population	Age-standardized disability prevalence of cataracts (%)	Disability prevalence of cataracts among a population ≥65 years (%)
Beijing	74,795	0.213	2.018
Tianjin	72,351	0.325	3.016
Heilongjiang Province	70,405	0.371	3.139
Liaoning Province	83,857	0.407	3.635
Shanghai Province	73,864	0.408	3.750
Inner Mongol Autonomous Region	65,247	0.476	4.327
Shanxi Province	75,016	0.488	4.486
Xinjiang Autonomous Region	51,775	0.494	4.497
Jilin Province	76,408	0.532	4.899
Hebei Province	104,713	0.566	5.274
Guizhou Province	104,169	0.568	5.353
Shaanxi Province	82,460	0.576	5.324
Zhejiang Province	75,636	0.600	5.645
Hunan Province	73,425	0.610	5.144
Chongqing	95,392	0.619	5.307
Jiangsu Province	106,065	0.620	5.851
Shandong Province	127,567	0.648	6.206
Hubei Province	101,674	0.713	6.448
Anhui Province	105,201	0.819	7.317
Henan Province	125,641	0.920	8.671
Qinghai Province	130,415	0.925	8.838
Jiangxi Province	83,088	0.937	8.811
Sichuan Province	30,395	0.965	8.460
Ningxia Autonomous Region	45,882	0.982	9.765
Guangdong Province	63,249	1.059	10.313
Gansu Province	41,214	1.064	10.160
Hainan Province	71,484	1.075	10.390
Fujian Province	125,442	1.076	10.423
Guangxi Autonomous Region	80,712	1.180	11.081
Yunnan Province	86,032	1.336	12.798
Xizang Autonomous Region	22,571	1.651	15.148
Average	74,795	0.749	6.984

## Results

### 1. Distribution of the disability prevalence of cataracts

The Second China National Sample Survey on Disability surveyed 2,525,145 people, and the total number with a visual disability caused by cataracts was 18,085. As shown in Table 2, the average age-standardized disability prevalence of cataracts in the country was 749 per 100,000. Beijing had the lowest age-standardized disability prevalence of cataracts at 213 per 100,000, while Xizang (Tibet) had the highest age-standardized disability prevalence of cataracts at 1,651 per 100,000. The national average age-standardized disability prevalence of cataracts among a population ≥65 years old was 6,984 per 100,000. The lowest disability prevalence of cataracts among a population ≥65 years old was observed in Beijing (2,018 per 100,000), while the highest was observed in Xizang (15,148 per 100,000).

### 2. Distribution of the ambient erythemal UVR

The lowest ambient erythemal UVR was recorded in Heilongjiang province with a daily exposure of 1728.94 J/m^2^ and an annual exposure of 631.06 KJ/m^2^. The highest ambient erythemal UVR was recorded in Xizang with a daily exposure of 4688.54 J/m^2^ and an annual exposure of 1776.29 KJ/m^2^. The annual ambient erythemal UVR of the surveyed regions in different decades are shown and sorted by the average ambient erythemal UVR in Table 3.

**Table 3:** Annual ambient erythemal UVR of 31 regions in different decades in China [sorted by the average ambient erythemal UVR (KJ/m^2^)].

Region	1980s	1990s	2000s	Average
Heilongjiang Province	603.87	652.66	636.66	631.06
Jilin Province	719.85	758.46	739.85	739.39
Liaoning Province	796.06	818.29	813.66	809.33
Inner Mongol Autonomous Region	818.05	862.52	854.91	845.16
Tianjin	871.57	872.80	862.74	869.04
Beijing	864.66	876.80	869.87	870.44
Hebei Province	893.34	907.27	896.31	898.97
Chongqing	944.77	920.81	929.89	931.82
Shandong Province	964.15	955.71	942.13	954.00
Henan Province	995.68	994.67	965.63	985.33
Jiangsu Province	1013.14	973.48	979.03	988.55
Hubei Province	1004.06	996.56	979.05	993.22
Shanxi Province	989.98	1012.00	988.64	996.87
Anhui Province	1017.54	988.30	984.92	996.92
Shaanxi Province	994.66	1019.63	1010.50	1008.27
Hunan Province	1062.59	1003.81	972.12	1012.84
Guizhou Province	1067.72	1004.04	991.32	1021.03
Shanghai	1057.67	1002.70	1002.86	1021.08
Zhejiang Province	1095.34	1034.29	1010.12	1046.58
Jiangxi Province	1117.50	1050.13	1021.50	1063.04
Ningxia Autonomous Region	1088.52	1113.60	1111.28	1104.47
Fujian Province	1189.63	1114.35	1095.88	1133.29
Guangxi Autonomous Region	1195.32	1108.11	1098.18	1133.87
Xinjiang Autonomous Region	1138.64	1178.44	1170.82	1162.63
Guangdong Province	1248.23	1178.36	1166.28	1197.63
Gansu Province	1200.5	1225.31	1216.96	1214.26
Sichuan Province	1426.37	1381.64	1380.06	1396.02
Hainan Province	1534.21	1414.96	1336.03	1428.40
Yunnan Province	1516.06	1397.98	1423.23	1445.75
Qinghai Province	1696.85	1696.97	1708.08	1700.63
Xizang Autonomous Region	2026.67	1977.06	1325.13	1776.29

### 3. Relationship between the disability prevalence of cataracts and the annual ambient erythemal UVR

**Figure 4:**
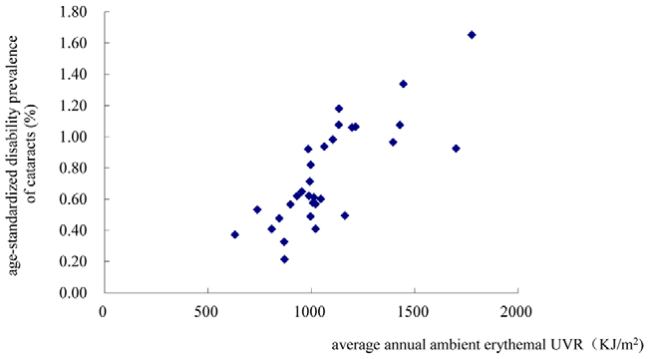
Scatter plot of the relationship between the age-standardized disability prevalence of cataracts (y) and the average annual ambient erythemal UVR (x) in 31 regions in China.

**Figure 5:**
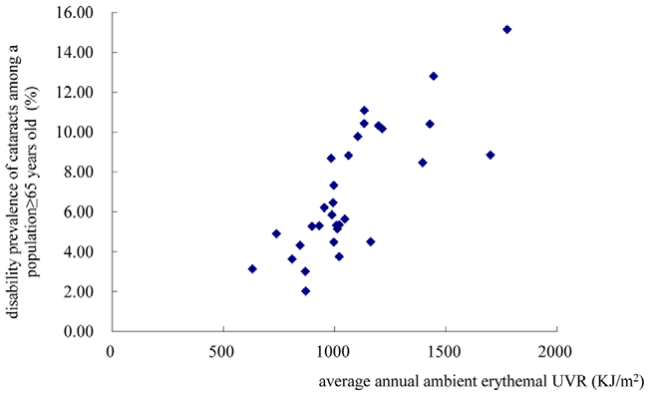
Scatter plot of the relationship between the disability prevalence of cataracts among a population ≥65 years old (y) and the average annual ambient erythemal UVR (x) in 31 regions in China.

As shown in Figures 4 and 5, the age-standardized disability prevalence of cataracts and the disability prevalence of cataracts among a population ≥65 years both increased with increases in the annual exposure to erythemal UV. The age-standardized disability prevalence of cataracts and the disability prevalence of cataracts among a population ≥65 years were found to be associated with the annual ambient erythemal UVR in the logistic regression models [OR_ age-standardized disability prevalence of cataracts_ = 3.97, 95%CI 1.30–12.13, per 100KJ/m^2^ increase in annual ambient erythemal UVR; OR_ disability prevalence of cataracts among a population ≥65 years_ = 3.97, 95%CI 1.30–12.13, per 100 KJ/m^2^ increase in annual ambient erythemal UVR].

### 4. The influence of latitude and altitude

As shown in Table 4, the median annual ambient erythemal UVR of latitude group 1 was 288.13 KJ/m^2^ higher than that of latitude group 3 (P = 0.005). Compared to latitude group 3, the median age-standardized disability prevalence of cataracts was 652/100,000 higher (P<0.001), and the median disability prevalence of cataracts among a population ≥65 years old was 6,678/100,000 higher (P<0.001) in latitude group 1. The median annual ambient erythemal UVR of latitude group 2 was 151.76 KJ/m^2^ higher than that of latitude group 3 (P = 0.002). Compared to latitude group 3, the median age-standardized disability prevalence of cataracts was 306/100,000 higher (P<0.001), and the median disability prevalence of cataracts among a population ≥65 years old was 2,813/100,000 higher in latitude group 2 (P<0.001).

**Table 4:** Comparison of the age-standardized disability prevalence of cataracts, the disability prevalence of cataracts among a population ≥65 years old and the ambient erythemal UVR among the three latitude groups.

Latitude group	n	Average latitude	Age-standardized disability prevalence of cataracts (%) Median	Disability prevalence of cataracts among a population ≥65 years old (%) Median	Annual ambient erythemal UVR (KJ/m^2^) Median
1	9	25.1°N	1.059*	10.313**	1133.29**
2	15	33.9°N	0.713*	6.448**	996.92*
3	7	43.4°N	0.407	3.635	845.16

* Difference is statistically significant compared to group 3, P<0.017.

** Difference is statistically significant compared to group 3, P<0.001.

As shown in Table 5, the medians of the age-standardized disability prevalence of cataracts and the medians of the disability prevalence of cataracts among a population ≥65 years old were not significantly different for any of the altitude groups. The median annual ambient erythemal UVR of altitude group 1 was 747.57 KJ/m^2^ higher than that of altitude group 3 (P = 0.009).

**Table 5:** Comparison of the age-standardized disability prevalence of cataracts, the disability prevalence of cataracts among a population ≥65 years old and the ambient erythemal UVR among the three altitude groups.

Altitude group	n	Average altitude (meters)	Age-standardized disability prevalence of cataracts (%) Median	Disability prevalence of cataracts among a population ≥65 years (%) Median	Annual ambient erythemal UVR (KJ/m^2^) Median
1	2	4000	1.288	11.993	1738.46
2	9	500–2000	0.576	5.353	1104.47
3	20	<500	0.619	5.748	990.89*

* Difference is statistically significant compared to group 1, P<0.017.

### 5. Influence of urbanization level and educational level

Age-standardized disability prevalence of cataracts and the disability prevalence of cataracts among a population ≥65 years old in Hebei Province were 566 per 100,000 and 5,274 per 100,000, respectively, while in Beijing, these values were 213 per 100,000 and 2,018 per 100,000, respectively. The disability prevalence of cataracts in Hebei was higher when compared to that in Beijing. The age-standardized disability prevalence of cataracts and the disability prevalence of cataracts among a population ≥65 years old in Zhejiang Province were 600 per 100,000 and 5,645 per 100,000, respectively, while in Shanghai, these values were 408 per 100,000 and 3,750 per 100,000, respectively. The age-standardized disability prevalence of cataracts and the disability prevalence of cataracts among a population ≥65 years old were higher in Zhejiang Province compared with Shanghai.

## Discussion

The results in the present study demonstrated that Xizang (Tibet) had the highest ambient erythemal UVR (annual exposure: 1776.29 KJ/m^2^) and the highest age-standardized disability prevalence of cataracts (1,651 per 100,000). Xizang has an average altitude of over 4,000 meters, which makes the air much thinner than in the lower, plains regions. Additionally, the long duration of sunshine and the intense reflection and scattering of UV radiation due to rocks and snow on the Xizang Plateau also contribute to the high ambient erythemal UVR in Xizang. Hainan Province (which is located at the southernmost part of China and has the lowest average latitude and a low altitude) also had a very high annual ambient erythemal UVR and a high disability prevalence of cataracts. Conversely, regions of the northeastern part of China (which has the highest latitudes, low altitudes, and shorter hours of sunshine) had the lowest ambient erythemal UVR and the lowest disability prevalence of cataracts.

Notably, the present study found that when we controlled the effect of age structure of populations by age-standardizing, there was a strong correlation between the age-standardized disability prevalence of cataracts and the disability prevalence of cataracts among a population ≥65 years old. The reason would be that cataract is generally a disease of older age and the age-standardized disability prevalence was largely made up of the disability prevalence of cataracts among a population ≥65 years old.

In the present study, we found that the age-standardized disability prevalence of cataracts and the disability prevalence of cataracts among a population ≥65 years old were both associated with the annual ambient erythemal UVR in 31 regions of China. A 100KJ/m^2^ increase in the annual ambient erythemal UVR was associated with a 397% increase in the log odds of having a regional age-standardized disability prevalence of cataracts or a disability prevalence of cataracts among a population ≥65 years above the median level.

Latitude and altitude are two main determinants of both ambient erythemal UVR level and disability prevalence of cataracts. Javitt and Taylor identified UVB (280–320 nm) exposure as inversely correlated with latitude and showed that the probability of cataract surgery in the U.S. increased by 3% for each 1-degree decrease in latitude [19]. Our findings also showed that the age-standardized disability prevalence of cataracts, the disability prevalence of cataracts among a population ≥65 years old, and the ambient erythemal UVR were significantly higher in low and middle latitude regions than in high latitude regions. Blumthaler et al. found that UVB irradiance increased with altitude in and over the UVB band: 24% per 1,000 m at 300 nm and 11% per 1,000 m at 320 nm inside the UVB band and 9% per 1,000 m at 370 nm(UVA 320–400 nm) outside the UVB band [20]. The present study showed that the ambient erythemal UVR in regions with an average altitude above 4000 m (including Xizang and Qinghai Provinces) were significantly higher than that in regions with an average altitude between 500–2000 m. However, the age-standardized disability prevalence of cataracts and the disability prevalence of cataracts among a population ≥65 years in the three altitude groups were not significantly different. The lack of significance in these results could be the small sample size in each altitude group.

Beijing and Shanghai had a lower age-standardized disability prevalence of cataracts and disability prevalence of cataracts among a population ≥65 years old compared to Hebei and Zhejiang, which are geographically similar to Beijing and Shanghai respectively, but with a much lower population density. The findings are thus consistent with a lower level of outdoor exposure in the more urbanized regions, and thus with the findings that higher UVR exposure (a combination of ambient UVR and time outdoors) increases the likelihood of having a disabling cataract.

Several strengths were presented in this study. First, this is the first study to investigate the relationship between the disability prevalence of cataracts and ambient erythemal UVR in China. A very large sample size from the Second China National Sample Survey on Disability provided this study with a good representation of people of color. Second, we included 31 regions of China in this study. A wide range in latitude and altitude in these regions provides a broad environmental gradient for estimating ambient erythemal UVR and the disability prevalence of cataracts. Third, vision disability not only devalues the quality of life of cataract patient, especially among the elderly, but also brings serious economic and disease burden to individuals, families and communities. The results of this study can provide scientific support to further research on the prevention of cataracts, especially for people of color, thus help to decrease the disease burden of cataracts and improve cataract patients' quality of life.

However, several limitations in the present study must be mentioned. First, we used the average ambient erythemal UVR in each region to describe the ambient UVR. It did not reflect the actual UV exposure of individual populations. The geographic environment and population distribution in some regions were so complicated that we may have masked the affect of these characteristics by using the average value. For example, the average altitude in the eastern part of Sichuan Province is below 500 meters, whereas the average altitude in the western part is between 4,000 and 4,500 meters. It is possible there was a substantial difference in ambient erythemal UVR between these two regions. Most of the population in Sichuan Province was located in the eastern part; thus, when we used the average ambient erythemal UVR in each region, we may have overestimated the actual UV exposure of the local population and masked the affect related to the differences in geographic characteristics and population density. Second, many factors influence the actual UV exposure of a population, such as activity time outdoors and air quality. To roughly reflect the influence of activity time outdoors on the disability prevalence of cataracts, we compared the disability prevalence of cataracts between regions with similar latitude and altitude (Beijing vs. Hebei; Shanghai vs. Zhejiang); however, precise estimation of the activity time outdoors of the population is difficult to achieve. Additionally, air quality (smog or dust) in urban areas could have a major effect on actual UV exposure. For example, the number of smog days in Beijing might have a major effect on individual UV exposure. Third, some factors that can influence the disability prevalence of cataracts, such as cataract surgery frequency, diabetes, medications and smoking were not included in the present study. For example, patients who had surgery would have normal vision and would not have been detected in the screen for disability. It is plausible, for example, that people who live in Beijing or Shanghai have much better access to cataract surgery and have a much higher volume of surgery performed. This would result in lower rates of visually disabling cataracts in these areas. Finally, studies have shown that UVR was mainly related to the prevalence of cortical cataracts [10]. However, the disability prevalence of cortical cataracts is difficult to obtain in 31 regions because the Second China National Sample Survey on Disability did not capture details on the types of cataracts. Although the survey was not designed for our study, the national data of disability prevalence was still very valuable in studying the health effects of increased ambient UVR.
